# ALK抑制剂在治疗NSCLC脑转移中的疗效及安全性研究进展

**DOI:** 10.3779/j.issn.1009-3419.2023.101.10

**Published:** 2023-05-20

**Authors:** CHEN Yuchen, HAN Han, WEI Jinpan, DU Qianyu, WANG Xiyong

**Affiliations:** 234000 宿州，安徽医科大学附属宿州医院（安徽省宿州市立医院）肿瘤科; Department of Oncology, Suzhou Hospital of Anhui Medical University (Suzhou Municipal Hospital of Anhui Province),Suzhou 234000, China

**Keywords:** 肺肿瘤, ALK抑制剂, 脑转移, 疗效, 安全性, Lung neoplasms, ALK inhibitors, Brain metastasis, Efficacy, Safety

## Abstract

肺癌是全球死亡率最高的恶性肿瘤之一，其中非小细胞肺癌（non-small cell lung cancer, NSCLC）占所有肺癌病理类型的80%-85%。NSCLC中有30%-55%的患者发生脑转移。据估计，5%-6%的脑转移患者存在间变性淋巴瘤激酶（anaplastic lymphoma kinase, ALK）融合。ALK融合阳性NSCLC患者在接受ALK抑制剂后获得了非常显著的疗效。经过十余年的迅速发展，ALK抑制剂已经形成三代同堂的局面：即第一代——克唑替尼（Crizotinib）；第二代——阿来替尼（Alectinib）、布格替尼（Brigatinib）、塞瑞替尼（Ceritinib）、恩沙替尼（Ensartinib）；第三代——洛拉替尼（Lorlatinib）。这些药物在ALK融合阳性NSCLC脑转移患者中显示出不同的疗效。由于此类药物众多，ALK抑制剂的选择给临床医生带来了困扰。因此，本文旨在对ALK抑制剂在NSCLC脑转移中的治疗效果和安全性进行综述，以期为临床医生提供治疗选择的依据。

## Abstract (en)

Lung cancer is one of the most lethal malignancies in the world, with non-small cell lung cancer (NSCLC) accounting for approximately 80%-85% of all pathological types. Approximately 30%-55% of NSCLC patients develop brain metastases. It has been reported that 5%-6% of patients with brain metastases harbor anaplastic lymphoma kinase (ALK) fusion. ALK-positive NSCLC patients have shown significant therapeutic benefits after treatment with ALK inhibitors. Over the past decade, ALK inhibitors have rapidly evolved and now exist in three generations: first-generation drugs such as Crizotinib; second-generation drugs including Alectinib, Brigatinib, Ceritinib, and Ensartinib; and third-generation drugs like Lorlatinib. These drugs have exhibited varying efficacy in treating brain metastases in ALK-positive NSCLC patients. However, the numerous options available for ALK inhibition present a challenge for clinical decision-making. Therefore, this review aims to provide clinical guidance by summarizing the efficacy and safety of ALK inhibitors in treating NSCLC brain metastases.

肺癌是世界范围内最常见的恶性肿瘤之一，而非小细胞肺癌（non-small cell lung cancer, NSCLC）患者在治疗期间发生脑转移的比例较高，为30%-55%。近年来，靶向药物的面世大大提高了患者的生存获益，成为NSCLC重要的诊疗方案。间变性淋巴瘤激酶（anaplastic lymphoma kinase, ALK）基因重排在2007年首次被发现，它存在于3%-7%的NSCLC患者中^[1]^。该突变是肺癌靶向治疗领域继表皮生长因子受体（epidermal growth factor receptor, EGFR）之后的又一重要发现。随着ALK通路的深入研究，各种ALK抑制剂层出不穷。克唑替尼（Crizotinib）是最早被研发出来的ALK抑制剂，故又称初代药。虽然相较于铂类，克唑替尼具有更优异的疗效，但在治疗开始后的12个月内频繁出现耐药现象^[2]^。第二代药物[阿来替尼（Alectinib）、布格替尼（Brigatinib）、塞瑞替尼（Ceritinib）、恩沙替尼（Ensartinib）]以及第三代药物[洛拉替尼（Lorlatinib）]已陆续上市，极大地丰富了用药格局（图1）。虽然第二代药物疗效有所提高，但脑转移和耐药等情况的发生仍不可避免。洛拉替尼独特的分子结构使其具有更高的选择性和中枢神经系统（central nervous system, CNS）渗透性，在第一、二代药物治疗失败的情况下，仍具有抗肿瘤活性^[3]^。目前，以NVL-655为代表的第四代药物正在紧锣密鼓地研制，初步的临床前试验^[4]^已表现出优越的抗肿瘤活性。本文就ALK抑制剂在ALK融合阳性NSCLC中的脑转移治疗效果和安全性进行分析，旨在为ALK阳性NSCLC脑转移患者提供最佳的治疗方案。

**图1:**
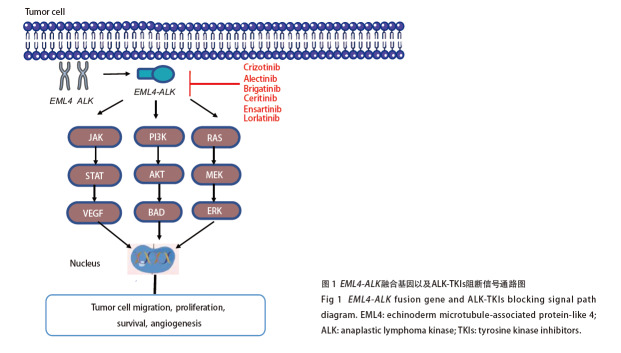
EML4-ALK融合基因以及ALK-TKIs阻断信号通路图

## 1 ALK基因突变

ALK是一种跨膜酪氨酸激酶单体，属于胰岛素受体超家族的一个成员^[5]^。研究^[6]^发现ALK基因突变是NSCLC中的驱动基因之一。其类型主要有三种，分别是ALK融合突变、扩增突变和点突变。其中最常见的ALK突变是棘皮动物微管相关蛋白样蛋白4-ALK（echinoderm microtubule-associated protein-like 4-ALK, EML4-ALK）融合突变。这一突变的表达方式是人类2号染色体短臂的ALK与EML4基因发生倒位融合，从而表达出新的蛋白。该突变可激活多种信号传导通路，如PI3K-AKT^[7]^、JAK-STAT^[8]^、mTOR、Ras-MEK^[9]^信号级联等信号传导通路，促进细胞的生长增殖、分化以及抗凋亡信号的发生（图1）。正因为EML4-ALK融合突变具有重要的临床意义^[10]^，检测该基因已成为肺癌个体化诊疗的指导依据。现在常用的检测ALK基因的方法是免疫组化（immunohistochemistry, IHC）、荧光原位杂交（fluorescence in situ hybridization, FISH）、二代测序（next-generation sequencing, NGS）等。其中，NGS具有高通量、高敏感性等优势，已经成为目前检测ALK突变的主流方法^[11]^。在肺癌术后的患者中，影像学检查常常无法及时发现肿瘤的复发，为了早期发现肿瘤复发转移的征象，循环肿瘤DNA（circulating tumor DNA, ctDNA）这一检测方法应运而生。该方法是通过检测血浆中脱落的DNA，发现ALK基因的突变^[12]^。

## 2 ALK抑制剂在NSCLC脑转移中的疗效

### 2.1 第一代ALK抑制剂

随着分子生物学的不断发展，各种ALK-酪氨酸激酶抑制剂（tyrosine kinase inhibitors, TKIs）已经问世。这些药物通过特异性地抑制ALK融合蛋白酪氨酸激酶活性，从而阻断信号传导通路，达到抗肿瘤的目的（图1）。第一个被开发出来通过抑制ALK发挥作用的药物是克唑替尼，因此也被称作第一代ALK抑制剂（表1）。它是一种小型分子制剂^[13]^。Profile1014研究^[14]^探讨了克唑替尼与化疗对ALK阳性NSCLC患者的疗效。该研究结果显示，克唑替尼组患者的客观缓解率（objective response rate, ORR）为74.0%，中位无进展生存期（median progression-free survival, mPFS）为10.9个月，显著优于化疗组（P<0.01）。基于这些重要的结果，美国食品药品监督管理局（Food and Drug Administration, FDA）批准了克唑替尼作为ALK阳性NSCLC患者的一线治疗。这些发现表明了克唑替尼在改善ALK阳性NSCLC患者治疗方面的潜力。同时，在该研究^[15]^的亚组分析中，针对基线23%的脑转移人群，克唑替尼组第12周颅内疾病控制率（disease control rate, DCR）是85.0%，比化疗组的45.0%显著增高（P<0.001）；第24周颅内DCR为56.0%，比化疗组的25.0%显著升高（P=0.006）。在基线脑转移患者中，两组mPFS分别是9.0个月 vs 4.0个月（P<0.001）；在基线无脑转移患者中，两组mPFS分别是11.1个月 vs 7.2个月（P<0.001）。总体而言，无论基线有无脑转移，克唑替尼相较于传统化疗都有更大的临床获益。因为克唑替尼对血脑屏障（blood-brain barrier, BBB）的透过率低，所以多数患者使用克唑替尼治疗后会发生耐药，以颅内病变进展最为常见。因此，急需开发出更多有效的药物用于临床患者。

**表1:** ALK阳性NSCLC治疗相关临床试验及结果

TKIs	Study	mPFS (mon)	mOS (mon)	ORR	Clinicial Trial.gov
Crizotinib vs CT	Profile1014	10.9 vs 7.0	NR vs 47.5	74.0% vs 45.0%	NCT01154140
Alectinib vs Crizotinib	ALEX	34.8 vs 10.9	NR vs NR	82.9% vs 75.5%	NCT02075840
	J-ALEX	NR vs 10.2	NR vs 43.7	85.0% vs 70.0%	JapicCTI-132316
Brigatinib vs Crizotinib	ALTA-1L	24.0 vs 11.1	NR vs NR	-	NCT02737501
Ceritinib vs CT	ASCEND-4	16.6 vs 8.1	NR vs 26.2	73.0% vs 2.0%-7.0%	NCT01828099
Ensartinib vs Cizotinib	eXalt-3	25.8 vs 12.7	NR vs NR	74.0% vs 67.0%	NCT02767804
Lorlatinib vs Crizotinib	CROWN	NR vs 9.3	NR vs NR	76.0% vs 58.0%	NCT03052608
TPX-0131	FORGE-1	-	-	-	NCT04849273
NVL-655	ALKOVE-1	-	-	-	NCT05384626

CT: platinum-based chemotherapy; mPFS: median progression-free survival; mOS: median overall survival; ORR: objective response rate; NR: not reached; NE: not estimable.

### 2.2 第二代ALK抑制剂

#### 2.2.1 阿来替尼

随着ALK抑制剂在NSCLC治疗中的应用越来越火热，第二代ALK抑制剂也逐渐被研发出来。其中一个首要目标是提高药物通过BBB的能力，使之更好地治疗脑转移。阿来替尼是第一款第二代ALK抑制剂（表1）。它采用了独特的苯并咔唑支架结构，能够与ALK激酶区完全适配结合，从而表现出高选择性^[16]^。此外阿来替尼绕开了P-糖蛋白这一重要的外排转运蛋白，BBB透过率明显提高，从而增强了药物的疗效。多项研究结果表明，阿来替尼治疗ALK阳性的NSCLC患者疗效良好。比如，Kodama等^[17]^开展的阿来替尼在颅内肿瘤临床前模型中的疗效研究，旨在观察阿来替尼对脑转移的疗效。研究结果表明，阿来替尼对颅内EML4-ALK融合阳性的患者效果良好，可为克唑替尼治疗的脑转移患者提供新的治疗机会（两组mPFS：105.2 d vs 39 d，P=0.0065）。而Hida等^[18]^开展的J-ALEX研究，旨在评估阿来替尼与克唑替尼治疗ALK阳性NSCLC患者的疗效。研究结果表明，相较于克唑替尼，阿来替尼则明显延长了患者的mPFS（未评估 vs 10.2个月，P<0.0001）。ALEX研究^[19]^在2018年公布的数据显示，无论基线有无脑转移，使用阿来替尼后两组mPFS（基线脑转移组：NR vs 7.4个月，P<0.0001；基线无脑转移人群：NR vs 14.8个月，P=0.0024）并未出现显著差异（P=0.36）。无论之前是否接受放疗，阿来替尼组的CNS ORR均优于克唑替尼组（接受放疗患者：85.7% vs 71.4%；未接受放疗患者：78.6% vs 40.0%）。由此可见，阿来替尼在预防和治疗CNS的进展方面均取得了优秀的表现。随后Mok等^[20]^研究指出：阿来替尼组的mPFS是34.8个月，总生存期（overall survival, OS）未成熟，5年OS率达到62.5%。一项真实世界研究^[21]^数据得出：当克唑替尼治疗失败后，使用阿来替尼能够获得更高的mPFS（P=0.043），比使用塞瑞替尼和布格替尼预后更好。此外，阿来替尼的抗肿瘤活性对于克唑替尼耐药突变中的ALK G1268A突变也表现出有效的作用^[22]^。虽然阿来替尼对有些耐药突变有效，但仍可能导致继发耐药突变的发生。因此，还需要研发更有效的药物应用于临床。

#### 2.2.2 布格替尼

布格替尼分子结构中的二甲基氧化磷结构增强了药物的高选择性和高渗透性，具有通过BBB到达颅内的能力，并可延缓脑转移的进展（表1）。一项III期临床试验（ALTA-1L）由Camidge等^[23]^开展，旨在评估布格替尼与克唑替尼在晚期ALK阳性NSCLC患者中的疗效。研究结果显示，与克唑替尼相比，布格替尼显著提高脑转移患者的mPFS（24.0个月 vs 5.5个月，P<0.0001）和OS（4年OS率分别是71.0%和44.0%，P=0.020），从而提升了整体的治疗价值。J-ALTA研究^[24]^是一项针对日本ALK阳性晚期NSCLC患者的II期前瞻性临床研究。入组的72例患者中，47例是阿来替尼耐药的患者。针对阿来替尼难治性人群中，布格替尼的ORR为34%，DCR为79%，mPFS为7.3个月。此外，在数据截止时，8例基线存在CNS转移患者的颅内客观缓解率（objective response rate, ORR）为25.0%。3例基线时发生G1202R突变的患者中有1例（33.0%）达到了部分缓解（partial response, PR），其余11例继发突变患者中有6例达到客观缓解（55.0%）。该研究结果表明布格替尼对阿来替尼耐药的脑转移患者具有活性，并对各种继发耐药突变具有抗肿瘤活性。Popat等^[25]^进行了一项III期临床试验（ALTA-3），旨在对比布格替尼与阿来替尼在治疗克唑替尼耐药的ALK阳性NSCLC患者的疗效。该研究目的是为患者选择合适的序贯治疗。然而，结果表明，在PFS方面，两者并无显著差异（19.3个月 vs 19.2个月，P=0.8672）。

#### 2.2.3 塞瑞替尼

塞瑞替尼是由诺华公司研发的第二代ALK抑制剂（表1），ASCEND-1^[26]^、ASCEND-2^[27]^研究显示，塞瑞替尼显示出令人振奋的抗肿瘤活性和颅内控制能力。ASCEND4研究^[28]^进一步评估了塞瑞替尼相比铂类化疗的疗效和安全性。结果表明，塞瑞替尼组的mPFS（16.6个月 vs 8.1个月，P<0.00001）较为良好。在基线有脑转移患者中，塞瑞替尼组mPFS达到10.7个月，颅内ORR达到46.3%；化疗组则分别为6.7个月和21.2%。这一结果证明塞瑞替尼不仅显著延长患者生存期，且对颅内转移灶控制强而有效。Chow等^[29]^开展的一项II期临床试验（ASCEND-7）是目前唯一将脑膜转移患者纳入研究的ALK系列试验，基线脑转移率达到100%。研究结果显示，经过塞瑞替尼治疗后，18例基线脑膜转移患者的ORR（16.7%）、DCR（66.7%）、mPFS（5.2个月）、OS（7.2个月）均有所改善。塞瑞替尼在治疗脑转移方面取得突破性进展，为ALK阳性NSCLC脑转移患者带来了另一种治疗选择。一项大型荟萃分析^[30]^结果表明，相较塞瑞替尼，阿来替尼治疗后CNS进展速度显著降低（2.3% vs 27.3%, P=0.029）。然而，一项临床前研究^[31]^结果表明，塞瑞替尼不但对克唑替尼耐药突变有效，而且对阿来替尼导致的V1180L、I1171T继发耐药突变也敏感。虽然塞瑞替尼能克服多种耐药突变，但仍无法克服G1202R、F117C突变。

#### 2.2.4 恩沙替尼

恩沙替尼是新一代ALK抑制剂，是我国自主研发的创新药物之一（表1）。它在治疗NSCLC方面具有显著的潜力，为我国NSCLC患者提供了更多的治疗选择。eXalt3研究^[32]^是一项全球多中心、开放标签的III期临床研究，共有290例患者入组并被随机分配至恩沙替尼组或克唑替尼组。结果显示，恩沙替尼组在PFS方面表现显著优异（25.8个月 vs 12.7个月，P<0.001）。在基线存在脑转移的患者中，恩沙替尼和克唑替尼的颅内ORR分别是63.6%和21.1%。无论是否存在基线脑转移，恩沙替尼组的mPFS都得到了延长（基线脑转移患者：11.8个月 vs 7.5个月，P=0.0480；基线无脑转移患者：未评估 vs 16.6个月，P=0.003）。eXalt3研究的结果为ALK阳性的NSCLC患者提供了新的治疗方案和选择。因此，恩沙替尼被认为是未来肺癌中具有巨大潜力的候选药物之一。

### 2.3 第三代ALK抑制剂

洛拉替尼与第一、二代ALK-TKIs的最大不同之处在于采用了大环酰胺结构（表1）。此种结构与ATP结合袋之间的亲和力更强，因而在立体空间构象上更为立体、紧密，从而使得洛拉替尼能够对抗多种ALK继发耐药突变。此外，洛拉替尼另一个独特之处是分子量较小，更易穿过BBB，可延缓脑转移的进展。CROWN研究^[33]^旨在评估洛拉替尼与克唑替尼在治疗晚期ALK阳性NSCLC患者的具体疗效，两组mPFS分别是：未达到 vs 9.3个月（P<0.01）。在接受洛拉替尼治疗的患者中，有71%达到颅内完全缓解。整体而言，洛拉替尼在治疗ALK阳性NSCLC方面的疗效较好，尤其是对颅内病灶的控制能力，相较于其他药物而言更为显著。在一项针对脑转移的亚组分析^[34]^中，对于基线存在脑转移的患者，洛拉替尼不仅能够显著改善mPFS（未达到 vs 7.2个月，P<0.0001），而且还能够延缓CNS进展累计发病率（两组12个月CNS累计发病率分别是：7% vs 72%）。同时，根据一项荟萃分析^[35]^，通过药物曲线下积分排名总和（surface under the cumulative ranking curve, SUCRA）评估，洛拉替尼在延长CNS转移患者的mPFS方面表现最佳（SUCRA：洛拉替尼>布格替尼>阿来替尼>恩沙替尼>克唑替尼>塞瑞替尼）。

### 2.4 第四代ALK抑制剂

虽然洛拉替尼在CNS转移患者的治疗效果在所有ALK-TKIs中居于领先位置，但是耐药问题仍然需要被认真考虑。因此，迫切需要一种不受其他ALK-TKIs耐药突变影响且CNS渗透高的药物。TPX-0131和NVL-655是新一代ALK-TKIs，两者都具有靶向双点突变的作用（表1）。TPX-0131是一种形态紧密的大环抑制剂，可与ATP结合袋较好结合。TPX-0131作为新一代ALK-TKIs，最大优势在于它是目前唯一对各种化合物突变均表现出广泛有效活性的TKIs，这种特性完全可以满足未来医学的需求。在临床前评估阶段，TPX-0131还表现出高水平的CNS渗透性，并且能够有效抑制野生型和突变型耐药突变的能力。目前，一项评估TPX-0131临床疗效的I期/II期临床试验（FORGE-1）^[36]^正在进行中。NVL-655是一种具有双突变活性的小分子抑制剂。一项Wistar Han大鼠试验^[37]^充分证明了NVL-655在动物模型中透过BBB的能力，为脑转移患者的治疗开辟了广阔前景。除了TPX-011、NVL-655之外，还有TPX-0005、LOXO-195、LOXO-101等第四代药物有望面世，这些药物的面世不仅为ALK阳性的NSCLC患者提供了更多的治疗选择，而且有望进一步提升其临床治疗效果。

## 3 安全性分析

在ALK-TKIs治疗方面，Profile1014研究^[14]^结果表明，患者对克唑替尼整体耐受性较好。3级-4级不良事件（adverse events, AEs）多为谷丙转氨酶/谷草转氨酶（alanine aminotransferase/aspartate aminotransferase, ALT/AST）增加（14%），通过调整药物剂量，使ALT/AST检验指标逐渐趋于正常。此外，有少数患者（1%）发生间质性肺疾病，导致治疗中断。虽然使用阿来替尼的患者大多数AEs发生率较低，但仍有7%的患者因AEs中断治疗，33%的患者需要减量或退出治疗^[38]^。布格替尼剂量为180 mg qd时，肌酸磷酸激酶（44%）、腹泻（37%）是较常见的AEs^[39]^。ALTA-1L研究^[23]^结果显示78%患者发生3级-5级AEs，其中44%患者需要减量，72%中断治疗，13%停止治疗。塞瑞替尼^[40]^的常见AEs包括腹泻（69%）、恶心（66%）和呕吐（51%）。而ASCEND-8研究^[41]^证明了随餐服用450 mg的塞瑞替尼可以显著降低胃肠道AEs的发生率。恩沙替尼最常见的3级AEs为皮疹。为了减少皮疹的发生概率，一般需要将剂量调低至225 mg左右^[42]^。洛拉替尼导致的AEs多为轻至中度，包括高胆固醇血症（82.4%）和高甘油三酯血症（60.7%）等^[43]^。与克唑替尼相比，洛拉替尼3级-4级AEs更为常见，主要是因为高脂血症的频发。7%患者因AEs导致治疗终止，4%患者发生严重的AEs。此外，虽然5%的患者发生了致命的AEs，但并未有因药物引起的死亡事件发生。因此，在临床应用ALK-TKIs治疗患者时，可根据患者情况采取剂量调整、对症治疗等措施缓解患者的症状，从而减少AEs的发生。

## 4 总结与展望

近年来，靶向治疗在肿瘤学领域的迅猛发展，为肿瘤患者带来了更有效的治疗手段和更优的生存获益。对于晚期ALK阳性NSCLC患者，第一代ALK抑制剂克唑替尼已成为标准一线治疗，并明显延长了患者的生存时间。然而，克唑替尼的主要缺点在于其高CNS转移发生率，这也促使下一代ALK抑制剂的蓬勃发展。虽然下一代ALK抑制剂整体疗效及脑转移控制效果显著，但仍不可避免出现继发耐药。目前，有关第二、三代ALK抑制剂相关耐药机制的研究较少，且缺乏头对头研究，导致后续治疗方案不明确。因此，临床上可以进行二次活检，根据不同的耐药机制进行精准靶向治疗。当前研究应积极探索联合治疗策略以克服ALK抑制剂所引起的获得性耐药。洛拉替尼可克服多种耐药突变，因此可作为二代药物耐药后的首选后续治疗方案。阿来替尼和布格替尼具有优越的颅内控制效果，并已成为临床推荐的标准一线治疗。因此，未来的研究方向不仅要着重研发具有更高CNS渗透性的ALK抑制剂以缓解颅内进展的速度，还要针对耐药机制开发更有效的治疗方案，在积极控制原发灶的同时减少脑转移的发生。期待有更多的研究能够进一步明确针对ALK阳性NSCLC脑转移患者的最佳序贯治疗方案，进一步延长患者的生存期。
